# C5a-Preactivated Neutrophils Are Critical for Autoimmune-Induced Astrocyte Dysregulation in Neuromyelitis Optica Spectrum Disorder

**DOI:** 10.3389/fimmu.2018.01694

**Published:** 2018-07-23

**Authors:** Paweł Piatek, Małgorzata Domowicz, Natalia Lewkowicz, Patrycja Przygodzka, Mariola Matysiak, Katarzyna Dzitko, Przemysław Lewkowicz

**Affiliations:** ^1^Department of Neurology, Laboratory of Neuroimmunology, Medical University of Lodz, Lodz, Poland; ^2^Department of General Dentistry, Medical University of Lodz, Lodz, Poland; ^3^Institute of Medical Biology, Polish Academy of Sciences, Lodz, Poland; ^4^Department of Immunoparasitology, Faculty of Biology and Environmental Protection, Institute of Microbiology, Biotechnology and Immunology, University of Lodz, Lodz, Poland

**Keywords:** astrocytes, neutrophils, complement system, glutamate, neuromyelitis optica spectrum disorder

## Abstract

Neuromyelitis optica spectrum disorder (NMOSD) is an autoimmune neuroinflammatory disease. In contrast to multiple sclerosis, autoantibodies against aquaporin-4 (AQP4) expressed on astrocytic end-feet have been exclusively detected in sera of NMOSD patients. Several lines of evidence suggested that anti-AQP4 autoantibodies are pathogenic, but the mechanism triggering inflammation, impairment of astrocyte function, and the role of neutrophils presented in NMOSD cerebrospinal fluid remains unknown. In this study, we tested how human neutrophils affect astrocytes in the presence of anti-AQP4 Ab-positive serum derived from NMOSD patients. An *in vitro* model of inflammation consisted of human astrocyte line, NMOSD serum, and allogenic peripheral blood neutrophils from healthy individuals. We showed evidence of pathogenicity of NMOSD serum, which by consecutive action of anti-AQP4 Abs, complement system, and neutrophils affected astrocyte function. Anti-AQP4 Ab binding astrocytes initiated two parallel complementary reactions. The first one was dependent on the complement cytotoxicity *via* C5b-9 complex formation, and the second one on the reverse of astrocyte glutamate pump into extracellular space by C5a-preactivated neutrophils. As a consequence, astrocytes were partially destroyed; however, a major population of astrocytes polarized into proinflammatory cells which were characterized by pathological glutamate removal from extracellular space.

## Introduction

Neuromyelitis optica spectrum disorder (NMOSD) is a central nervous system (CNS) inflammatory autoimmune illness characterized by medullary and/or optical nerve damage leading to paralysis and blindness (1). For many years, NMOSD was believed to be a rare variant of multiple sclerosis (MS). In contrast to MS, autoantibodies against aquaporin-4 (anti-AQP4 Ab), a water channel densely expressed on astrocytic end-feet, have been detected in the majority of NMOSD cases, providing evidence of an autoimmune character of this disease and suggesting the need for another treatment approach (2). In addition, a specific profile of serum peptide reactivity to human antigens might be helpful in differentiating NMOSD and MS (3).

Recently, two different hypotheses explaining the consequences of anti-AQP4 Ab binding astrocytes were proposed (4). The first one highlighted the role of complement system activation during anti-AQP4 Ab binding which assumed that after crossing or direct at the blood–brain barrier (BBB), anti-AQP4–IgG binds extracellular conformation domains of aquaporin-4 (AQP4) and activates classical complement cascade causing deposition of the membrane attack complex C5b-9 into astrocyte cell membrane (5–9). Another theory postulated that anti-AQP4–IgG binding might affect astrocyte metabolism leading to disruption of their ability to neutralize extracellular glutamate. High extracellular concentration of glutamate is cytotoxic for CNS (10). Astrocytes are the major cells that regulate extracellular glutamate concentration, downregulating it, when it is briefly increased at glutamergic synapses and maintaining low extracellular level at rest (11).

Another aspect of anti-AQP4–IgG binding astrocytes deserving consideration is represented by functional changes of affected astrocytes that result in reactive astrocytosis. Astrocytes were shown to undergo various alterations in response to different types of injuries or diseases at morphological and biochemical levels (12). Reactive astrocytes are considered to be a cellular component of resident innate immune system in the CNS, which stand on guard protecting against microbial infections (13). Furthermore, activated astrocytes were shown to express and secrete chemokines and cytokines which might promote the recruitment of peripheral immune cells (14).

Although it has long been known that neutrophils are present in NMOSD CNS lesions and cerebrospinal fluid (CSF), their role in NMOSD pathogenesis has not been determined (15–17). Complement-dependent granulocyte recruitment through BBB as a result of anti-AQP4 Ab binding astrocytes has been described in *in vitro* experiments (6). In mouse models of NMOSD, the extent of CNS tissue damage correlated with neutrophil infiltration, and inhibition of neutrophil protease reduced anti-AQP4 Ab-induced brain tissue damage (18, 19).

In this study, we tested how neutrophils contributed to the NMOSD pathogenesis, providing evidence of their direct involvement in dysregulation of astrocyte function by mediating the formation of reactive astrocytes which possessed proinflammatory capacity and impaired glutamate metabolism.

## Patients and Methods

### Patients

All participants of the study were diagnosed and recruited at the Department of Neurology, Medical University of Lodz. Seven patients (four females, three males; aged 45.7) were diagnosed with NMOSD, fulfilling the 1999 Wingerchuk criteria for NMOSD (20). All the patients had optic nerve and spinal cord inflammation, none of them had brain magnetic resonance imaging that fulfilled Barkhof/Tintore criteria for MS, all were anti-AQP4 autoantibody positive (21). CSF and peripheral blood were collected at 3.4 ± 2.33 months from the first relapse. Two groups of patients were recruited as the controls. The first one constituted of the patients with remitting–relapsing multiple sclerosis (RRMS), diagnosed according to the McDonald criteria (22), representing neurodegenerative disease mainly dependent on autoreactive T lymphocytes. For NMOSD and RRMS, a relapse episode was defined as the appearance of new neurological signs or worsening of pre-existing signs after at least 30 days of clinical stability, while remission as a clinical stability for more than 3 months after the relapse. Both NMOSD and RRMS patients were seronegative for myelin oligodendrocyte glycoprotein (anti-MOG) autoantibodies. The second control group constituted of the patients with other neurological disorders (OND) characterized by negative medical history for autoimmune and infectious diseases. Eight patients that have been qualified to OND group suffered from headache and were negative for recognition of infectious meningitis and brain inflammation with negative clinical and CSF laboratory criteria: normal values of glucose (60–75% of serum concentration), total protein (0.15–0.45 g/L), Cl^−^ (117 mmol/L), and lactic acid (<2.1 mmol/L). All patients were treatment-naïve, none received systemic steroids or other anti-inflammatory drugs for at least 3 months before the study. The patients in the control groups were age and gender matched. Mean age was 43.2 and 41.5 for RRMS and OND patients, respectively. 2 mL of CSF, 10 mL of whole blood on lithium heparin anticoagulant, and 5 mL without anticoagulant were derived from all patients during routine testing. Six age- and gender-matched healthy volunteers were recruited to the study. 10 mL of whole blood on lithium heparin anticoagulant has been collected form each healthy control (HC).

The study was approved by the Ethics Committee of the Medical University of Lodz (RNN/44/14/KE), and informed consent was obtained from each participant of the study.

### Detection of Anti-AQP4 and Anti-MOG Serum Autoantibodies

Detection of serum anti-AQP4 and anti-MOG autoantibodies was done using Indirect Immunofluorescence Kits (Euroimmune, Lübeck, Germany) according to the manufacturer’s instruction.

### Multiple Profiling Chemokine Assays

Concentrations of chemokines and cytokines in CSF were measured using Bio-Plex Pro™ Human Chemokine Assays (Bio-Rad Laboratories). To analyze reactivity of astrocytes exposed to CSF and neutrophils, concentration of 27 pro- and anti-inflammatory cytokines and chemokines was measured in supernatants using Human Cytokine Group I Assays. All reagents and technology were provided by Bio-Rad Laboratories (Bio-Plex 200). Analysis was performed according to the manufacturer’s recommendations. Briefly, standards and samples were diluted (1:4) in sample diluent and transferred to the plate containing magnetic beads and incubated for 1 h at room temperature (RT). Next, the plate was washed (3×), and detection antibody was added for 30 min on shaker (850 rpm) at RT. Next, the plate was washed (3×), and streptavidin-PE solution was added for 10 min. After incubation, plate was washed (3×), and samples were resuspended in 125 µL of assay buffer and analyzed within 15 min. All samples were analyzed at the same time in duplicate wells.

### ELISA

C5a and glutamate were measured in CSF and supernatants using ELISA Kits (LifeSpan Biosciences and Abnova). The detection limit was 31.25 pg/mL for C5a and 0.3 µg/mL for glutamate. According to the manufacturer’s information, glutamate cross-reactivity with factors present in the culture media and CSF (glutamine, glycine, alanine, and 5-aminovaleric acid) was less than 0.01%. All samples were analyzed in duplicate.

### Lactate Dehydrogenase (LDH) Release Assay

Lactate dehydrogenase release was measured by colorimetric method using the Cytotoxicity Detection Kit (Sigma-Aldrich, St. Louis, MO, USA; Roche) according to the manufacturer’s instruction. Concentration of LDH released from astrocytes lysed with Triton X-100 solution (final concentration 1%) was used as the positive control (100% of cell lysis). Supernatant from astrocytes supplemented with 10% PBS was used as a negative control (spontaneous LDH release). The rate of lysed cells was calculated based on the normalization of each sample to the level of LDH released by positive and negative controls samples. All samples were analyzed in duplicate.

### Neutrophil Isolation

Neutrophils were isolated on Polymorphprep™ (Axis Shield) according to the manufacturer’s instruction. Purity and viability of isolated neutrophils was ~97% (Figure S1A in Supplementary Material).

### Adhesion Molecule Expression and Functional Tests of Neutrophils

#### *In Vitro* Study

Adhesion molecule expression, phagocytosis, and reactive oxygen intermediate (ROI) production were measured in neutrophils isolated form HCs after incubation with CSF for 30 min in 37°C. Unconjugated anti-C5a (1 µg/mL, clone 2942; Thermo Fisher Scientific) and mouse IgG2aκ (eBioscience™) as isotype control mAbs were used for neutralizing experiments.

#### *Ex Vivo* Study

Adhesion molecule expression, phagocytosis, and ROI production were measured within 30 min in freshly drowned neutrophils from NMOSD, RRMS, and OND patients.

### CD11b, CD18, and CD62L Expression

3 × 10^6^ cells/mL suspended in PBS were incubated at RT with conjugated monoclonal antibodies: anti-CD11b PE (ICRF44, BD), CD18-FITC (L130, BD), and CD62L-PE (SK11, BD). After 30 min of incubation, samples were washed in PBS, fixed with 1% paraformaldehyde, and analyzed using flow cytometry (BD LSRII, FACSDiva™ analysis software).

### Phagocytosis and ROI Production

Ready to use kits were used for analysis of neutrophil phagocytosis and ROI production (Bursttest and Phagoburst, OrphoGen Pharma, Germany). Measurements of peripheral blood neutrophil function were performed in the whole blood according to the manufacturer’s instruction, whereas experiments, in which CSF effect on neutrophils was analyzed, were performed in isolated cells. Samples were analyzed within 30 min using flow cytometry (BD LSRII, FACSDiva™).

### Chemotaxis Assay

Chemotaxis was determined with modified Migratest™ (Orpegen Pharma, Germany). Shortly, CSF was used as the medium contained unknown chemotaxis factors. Freshly isolated HC neutrophils were incubated in upper chamber (cell cultured insert with 3.0 µm ϕ) immersed in CSF (low chamber) for 30 min in 37°C. Neutrophils that have migrated through cell culture insert toward CSF or supernatant were labeled with propidium iodate, CD62L-FITC (fluorescein isothiocyanate) (BD) and analyzed within 30 min using flow cytometry (BD LSRII, FACSDiva™). The number of neutrophils was normalized using tubes containing constant number of microbeads (Trucount™, BD). As a positive chemotaxis control, *N*-formyl-methionyl-leucyl-phenylalanine (2 × 10^−6^ M) was used. PBS was used as a negative control.

### Human Astrocyte Culture

Normal cells derived from human induced neural stem cells (CL 05004-CLTH/astrocytes; Celther Poland, first passage 1 × 10^6^ cells/vial) after thawing under standard conditions were cultured in DMEM High Glucose medium (Sigma-Aldrich) supplemented with 1% fetal bovine serum (Sigma-Aldrich), 1% N2 supplement (Gibco Life Technologies), 100 U/mL penicillin and 0.1 mg/mL streptomycin (Sigma-Aldrich), and 100 ng/mL of BMP-4 supplement (TGFβ superfamily ligand, Merck) in T-25 flask coated by previously prepared Geltrex^®^ LDEV-Free reduced growth factor basement membrane matrix, according to the manufacturer’s protocol (Gibco Life Technologies) at 37°C and 5% CO_2_ (23). CLTH/astrocyte cultures were monitored everyday under a phase contrast microscope for 7 days with replacing the medium two times per week. For cytokine, C5a, glutamate, LDH release, ICC, and neutrophil chemotaxis assay, CLTH/astrocytes (second passage) were seeded at 2 × 10^4^/100 μL/well in U-bottom microtiter plates (Falcon, Becton Dickinson) and Cell Culture Chamber slide (Nalge Nunc International) coated by previously prepared Geltrex^®^ LDEV-Free reduced growth factor basement membrane matrix (Gibco Life Technologies). Cells were incubated for 24 h at 37°C in humidified atmosphere with 5% CO_2_ prior exposition to sera. More than 99% of cells were positive for glial fibrillary acidic protein (GFAP), a specific marker for astrocytes (Figure S1A in Supplementary Material).

### Immunocytochemical Analysis

Astrocytes were transferred to gelatin-coated microscope slides by cytospin (300 × *g*, 10 min) and fixed with 4% paraformaldehyde for 20 min at RT. Fixed cells were washed extensively with PBS and blocked with 10% rabbit normal blocking serum (Santa Cruz Biotechnology) supplemented with 0.3% Triton™ X-100 (Sigma-Aldrich) for 45 min at RT. Next, cells were washed and double-stained for IgG1 with AQP4, C5b-9 with phalloidin, and GFAP with C5b-9. Anti-IgG1/FITC (Dako Cytomation), anti-AQP4 (Santa Cruz Biotechnology), anti-C5b-9 (Dako Cytomation), phalloidin (Invitrogen), anti-GFAP (Santa Cruz Biotechnology), and rat IgG2b (Invitrogen), as negative isotype control were used. Secondary fluorescent antibodies [chicken pAb to rabbit Texas Red (TR) (Santa Cruz Biotechnology), goat pAbs to mouse FITC (Abcam), or goat pAbs to mouse TR (Thermo Fisher Scientific)] were added. The membrane localization of C5b-9, IgG1, and GFAP was analyzed by confocal microscopy (Nikon D-Eclipse C1) using EZ-C1 software.

### *In Vitro* Model of Induction of NMOSD Inflammation

Matured astrocytes were cultured as described above. First, anti-AQP4 Ab-positive NMOSD serum (active stage of the disease) was added to astrocyte culture for 1 h (10% v/v). Relapse RRMS or OND sera were used as the controls. Cells were washed and then incubated for 4 h with freshly isolated neutrophils of HCs. Next, neutrophils were removed by anti-CD15 magnetic MicroBeads (Miltenyi Biotec), and astrocytes were washed and cultured for additional 16 h. Supernatants were collected at three time points: baseline, after incubation with serum (1 h), and after incubation with neutrophils (21 h). To visualize astrocyte morphology, cells were cultured on 8-well chamber slides (Nunc International) and analyzed by imaging in DIC microscopy (Zeiss Axiovert 200 inverse microscope with a Zeiss Plan-apochromat 63×/1.43 differential interference contrast objective).

### Statistics

A statistical analysis of differences was performed using the one-way ANOVA test. Scheffe’s test was used for multiple comparisons as a *post hoc* test when statistical significances were identified in the ANOVA test. Statistical significance of differences in *ex vivo* study of neutrophil function and concentration of chemotaxis agents in CSF was determined by Student’s *t*-test.

## Results

### C5a Level Is Increased in the NMOSD CSF

**Figure 1:**
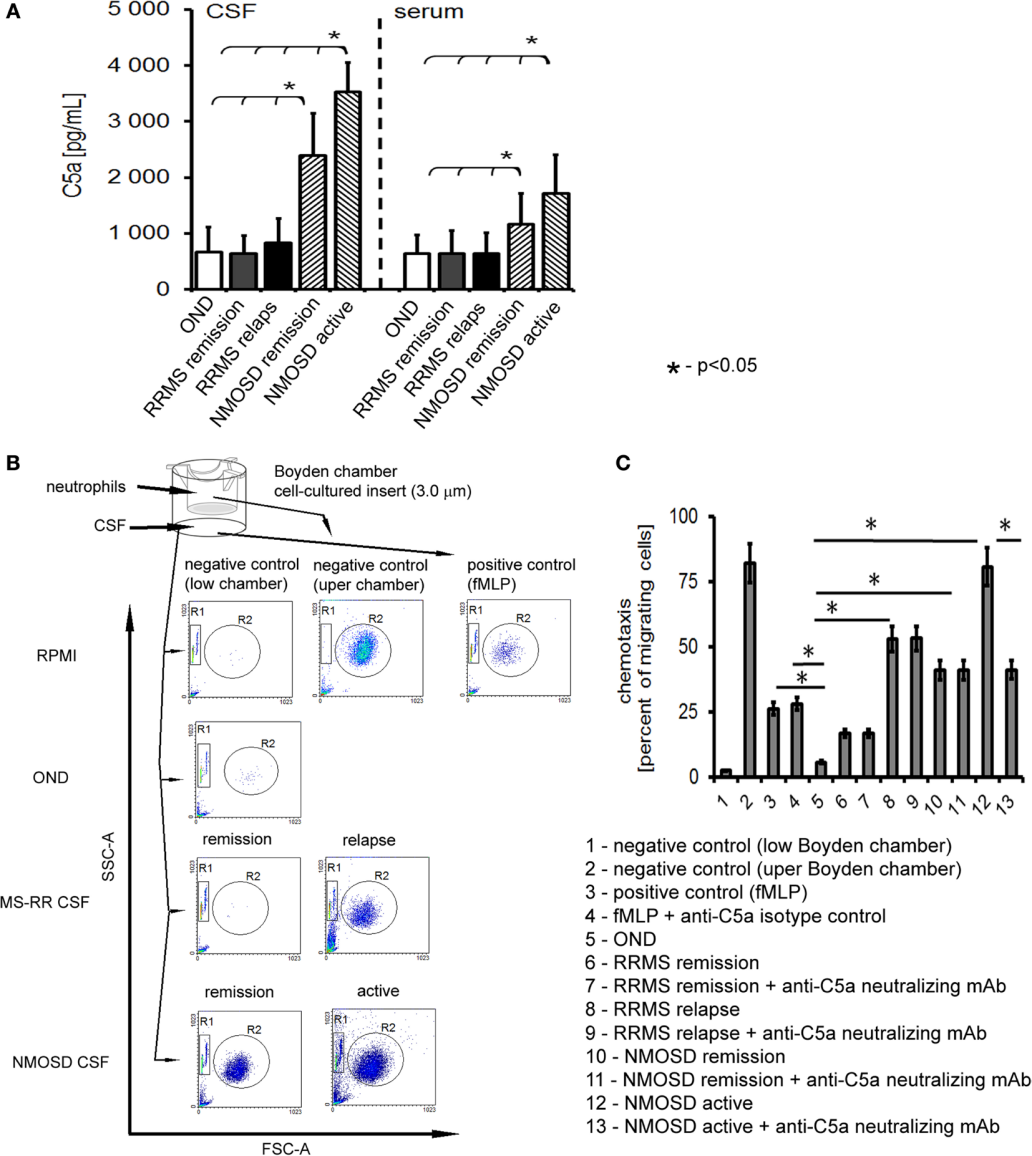
Cerebrospinal fluid (CSF) derived from neuromyelitis optica spectrum disorder (NMOSD) patients induces chemotaxis of healthy control neutrophils by C5a. **(A)** NMOSD is characterized by the high concentration of C5a in CSF and serum compared with remitting–relapsing multiple sclerosis (RRMS) or other neurological disorders (OND). The bars represent the means ± SD. **(B)** NMOSD CSF possesses chemotaxis properties independently of whether it was collected in the active or remission stage of the disease, while RRMS CSF possesses chemotaxis activity during disease relapse only. As a negative control, RPMI and neutrophils from upper chamber were used; as a positive control, fMLP was used. The example of one of four independent experiments. **(C)** NMOSD CSF chemotaxis properties are mainly dependent on the classical pathway of complement activation, as C5a neutralization inhibits neutrophil chemotaxis in NMOSD patients in oppose to RRMS CSF. Data are presented as the mean rates of cell migration ± SD from four independent experiments.

In the first phase of the study, concentrations of all well-documented endogenous chemokines responsible for neutrophil recruitment to the site of inflammation were analyzed. We used multi ELISA method that allowed to analyze many chemotaxis agents in one sample. Surprisingly, the majority of conventional chemotactic factors were not increased neither in the active nor in the remission NMOSD CSF (Table 1). Concentrations of inflammatory induced chemokines, such as ENA-78, Eotaxin-1-3, GRO-α, and GRO-β, were not statistically significantly different in NMOSD compared with both control groups. GCP-2 (CXCL6) level was below the detection limit (<12.0 pg/mL) in all studied groups. However, the level of IL-8, one of the most potent neutrophil chemotaxis factors was increased both in the active and remission NMOSD CSF compared with the control groups. Moreover, chemokines associated with recruitment of lymphocytes or monocytes/macrophages BCA-1, MIG, MIP-1α, and MIP-1δ were increased in the active and remission NMOSD CSF compared with RRMS CSF during relapse or remission. These data suggest that IL-8 might be responsible for neutrophil recruitment to CSF in NMOSD. C5a, which is released during activation of complement classic pathway, is recognized as a mediator of inflammation and another potent attractant and neutrophils activator (24). Consequently, we analyzed C5a level in CSF and serum and found that it was significantly increased in NMOSD (both in remission and active stage), contrary to RRMS or OND (Figure 1A).

**Table 1:** Cerebrospinal fluid (CSF) chemokine and cytokine concentrations in the patients with neuromyelitis optica spectrum disorder (NMOSD), remitting–relapsing multiple sclerosis (RRMS), and other neurological disorders (OND) (mean ± SD).

pg/mL	NMOSD active (*n* = 4)	NMOSD remission (*n* = 3)	OND (*n* = 8)	RRMS relapse (*n* = 4)	RRMS remission (*n* = 4)	*p* < 0.05
^†^6Ckine (CCL21)	175.7 ± 28.12	154.5 ± 31.77	185.3 ± 84.47	96.8 ± 67.36	167.5 ± 44.87	
^†^BCA-1 (CXCL13)	34.8 ± 33.39	23.0 ± 23.03	0.6 ± 0.27	4.2 ± 6.57	6.6 ± 6.67	a, d
^†^CTACK (CCL27)	3.9 ± 1.55	2.9 ± 1.06	2.9 ± 1.07	2.0 ± 0.73	4.5 ± 3.36	
^#^,^‡^ENA-78 (CXCL5)	59.3 ± 39.88	49.0 ± 38.90	68.0 ± 57.33	167.8 ± 78.51	98.1 ± 31.93	b, c
^#,†^Eotaxin-1 (CCL11)	11.8 ± 7.87	8.0 ± 6.29	4.6 ± 3.51	9.9 ± 6.01	6.9 ± 2.46	
^#,†^Eotaxin-2 (CCL24)	8.7 ± 7.66	1.3 ± 2.92	0.26 ± 0.000	0.3 ± 0.21	0.3 ± 0.02	
^#,†^Eotaxin -3 (CCL26)	5.1 ± 3.11	3.0 ± 0.99	2.0 ± 1.23	1.8 ± 0.53	2.0 ± 0.63	
^†,‡^Factalkine (CX3CL1)	80.9 ± 44.85	90.9 ± 41.09	71.2 ± 23.58	69.6 ± 33.82	81.6 ± 8.68	
^#,‡^GCP-2 (CXCL6)	<12.0	<12.0	<12.0	<12.0	<12.0	
^#,‡^GM-CSF	2.2 ± 2.08	1.0 ± 1.33	1.0 ± 1.15	1.1 ± 0.74	0.9 ± 0.65	
^#,‡^GRO-α (NAP-3 CXCL1)	28.3 ± 7.55	25.9 ± 7.90	19.8 ± 2.39	21.6 ± 1.61	28.7 ± 16.97	a, d
^#,‡^GRO -β (CXCL2)	2.7 ± 0.90	1.9 ± 0.42	1.3 ± 0.77	1.7 ± 0.52	1.9 ± 0.76	
^†,‡^I-309 (CCL1)	20.0 ± 7.39	17.7 ± 7.13	5.4 ± 2.46	9.5 ± 7.32	10.7 ± 4.16	a, b, d
IFN-γ	0.91 ± 0.410	0.27 ± 0.34	0.13 ± 0.035	0.14 ± 0.029	0.15 ± 0.034	a
IL-1β	7.6 ± 0.98	6.0 ± 0.88	0.50 ± 0.146	0.76 ± 0.235	1.20 ± 8.556	a, b, d
IL-2	0.83 ± 0.661	0.49 ± 0.456	0.41 ± 0.100	0.45 ± 0.070	0.49 ± 0.097	
IL-4	3.09 ± 1.777	2.65 ± 0.909	3.39 ± 2.296	6.88 ± 3.265	3.96 ± 1.850	
IL-6	7.0 ± 4.33	7.6 ± 2.69	8.9 ± 9.95	8.0 ± 10.11	11.3 ± 18.96	
^#,‡^IL-8 (CXCL8)	44.9 ± 23.99	36.8 ± 25.33	20.8 ± 7.27	14.5 ± 9.29	15.8 ± 20.67	b, c
IL-10	1.9 ± 1.03	18.6 ± 8.28	1.96 ± 0.679	3.02 ± 0.950	3.03 ± 1.674	b, c, d, e
IL-16	155.5 ± 108.77	97.0 ± 69.09	35.7 ± 19.56	45.4 ± 23.72	65.1 ± 33.69	
^†^IP-10 (CXCL10)	220.1 ± 205.44	146.7 ± 199.87	24.1 ± 12.29	29.9 ± 27.63	42.4 ± 21.40	
^†^I-TAC (CXCL11)	26.9 ± 25.80	28.3 ± 24.95	7.9 ± 3.18	12.9 ± 9.12	15.6 ± 7.35	
^†,‡^MCP-1 (CCL2)	169.3 ± 126.49	132.5 ± 112.59	111.1 ± 21.74	101.7 ± 55.76	125.7 ± 120.66	
^†,‡^MCP-2 (CCL8)	12.9 ± 13.90	8.0 ± 9.29	2.3 ± 1.65	2.1 ± 1.90	3.7 ± 0.95	
^†,‡^MCP-3 (CCL7)	8.7 ± 8.89	5.2 ± 4.02	1.7 ± 0.59	1.7 ± 0.63	1.9 ± 0.76	
^†,‡^MCP-4 (CCL13)	39.4 ± 41.54	31.9 ± 52.05	100.14 ± 10.37	50.1 ± 54.59	57.9 ± 52.48	a, d
^†^MDC (CCL22)	23.9 ± 30.03	13.9 ± 25.12	2.5 ± 1.02	7.5 ± 9.64	6.8 ± 5.81	
MIF	997.2 ± 979.97	699.9 ± 893.42	801.1 ± 501.02	223.7 ± 80.45	1,454.0 ± 2,957.27	
^†^MIG (CXCL9)	238.9 ± 209.54	169.9 ± 195.94	10.6 ± 3.78	17.7 ± 19.39	16.1 ± 4.71	a, b, c, d
^†,‡^MIP-1α (CCL3)	4.9 ± 3.90	3.0 ± 2.99	0.7 ± 0.24	1.9 ± 1.71	2.4 ± 0.83	a, d
^‡^MIP-1δ (CCL15)	207.1 ± 98.46	139.2 ± 93.29	85.9 ± 28.49	75.1 ± 26.93	119.5 ± 45.59	a, b
^†^MIP-3α (CCL20)	0.65 ± 0.497	0.38 ± 0.383	0.38 ± 0.076	0.46 ± 0.088	0.64 ± 0.557	
^†^MIP-3β (CCL19)	90.6 ± 59.98	65.2 ± 67.92	33.1 ± 13.07	34.2 ± 31.94	47.5 ± 27.61	a
^†,‡^MPIF-1 (CCL23)	55.1 ± 59.90	27.8 ± 21.88	86.0 ± 37.02	100.0 ± 80.00	58.9 ± 51.31	
^†^SCYB16 (CXCL16)	469.1 ± 43.19	449.1 ± 43.18	420.7 ± 35.64	404.6 ± 62.92	449.2 ± 29.52	
^†,‡^SDF-1α + β (CXCL12)	434.4 ± 243.71	399.8 ± 245.79	394.4 ± 193.50	212.0 ± 161.03	381.9 ± 155.81	b
^†^TARC (CCL17)	95.9 ± 67.98	53.9 ± 55.92	100.0 ± 0.00	100.00 ± 0.000	85.9 ± 37.23	
^†^TECK (CCL25)	77.8 ± 32.51	52.5 ± 29.55	45.3 ± 8.11	53.6 ± 11.93	61.1 ± 38.13	a, d
TNFα	12.6 ± 7.18	7.9 ± 4.54	4.2 ± 2.93	6.9 ± 3.89	6.3 ± 2.17	a, b, e

*Statistically significant differences between (a) NMOSD active and OND; (b) NMOSD active and relapse RRMS (c); NMOSD remission and remission RRMS; (d) NMOSD remission and OND; and (e) NMOSD active and NMOSD remission*.

*^#^Chemokine associated with neutrophil recruitment/activation*.

*^†^Chemokine associated with lymphocyte recruitment/activation*.

*^‡^Chemokine associated with monocyte/macrophage recruitment/activation*.

### C5a in NMOSD CSF Is Responsible for Neutrophil Chemotaxis, Diapedesis, and Preactivation to More Intensive Phagocytosis and ROI Production

We verified whether C5a present in CSF of NMOSD patients had an ability to induce chemotaxis of neutrophils. In this set of experiments, we used HC neutrophils to mimic all the pathophysiological mechanisms responsible for transition from health to pathology. We found that NMOSD CSF in the active and remission phase, as well as CSF from relapse MS, contrary to remission MS and OND, possessed chemotaxis properties toward non-stimulated HC neutrophils (Figure 1B). Deactivating C5a abrogated chemotaxis in the presence of NMOSD CSF by nearly 40%, compared with isotype control, but not in relapse MS CSF (Figure 1C). Analysis of the effect of NMOSD CSF on the surface adhesion molecules of HC neutrophils showed increased CD11b, CD18, and CD62L expression, contrary to MS or OND CSF. Experiments with neutralizing mAbs showed that this effect was dependent on the C5a, confirming its crucial role in preactivation of non-stimulated HC neutrophils (Figure S2A in Supplementary Material). In addition, this finding was confirmed by *ex vivo* analysis of circulating neutrophils from active NMOSD patients which were characterized by high expression of CD11b, CD18, and CD62L, opposite to RRMS, OND, or HC (Figure S2B in Supplementary Material).

Preactivated neutrophils are characterized by more intensive phagocytosis and ROI production. It is dependent on endo- and exogenous signals from the site of inflammation. We noted increased phagocytosis and ROI production in HC neutrophils preincubated with active NMOSD CSF and subsequently stimulated with *E. coli* (phagocytosis) or PMA (ROI production), contrary to RRMS or OND CSF (Figures S3A and S4A in Supplementary Material). This effect was reversed by C5a-neutralizing mAbs (Figures S3B and S4B in Supplementary Material). *Ex vivo* analysis of circulating neutrophils from active NMOSD patients showed more intensive phagocytosis and ROI production, compared with neutrophils from RRMS, OND, or HC (Figures S3C and S4C in Supplementary Material). This set of experiments revealed that NMOSD CSF preactivates HC neutrophils predominantly with C5a and this process is a reflection of neutrophil preactivation observed in NMOSD patients *in vivo* during the active phase of the disease.

### Anti-AQP4 Ab-Positive Serum Activates Membrane Attack Complex on the Surface of Astrocytes

**Figure 2:**
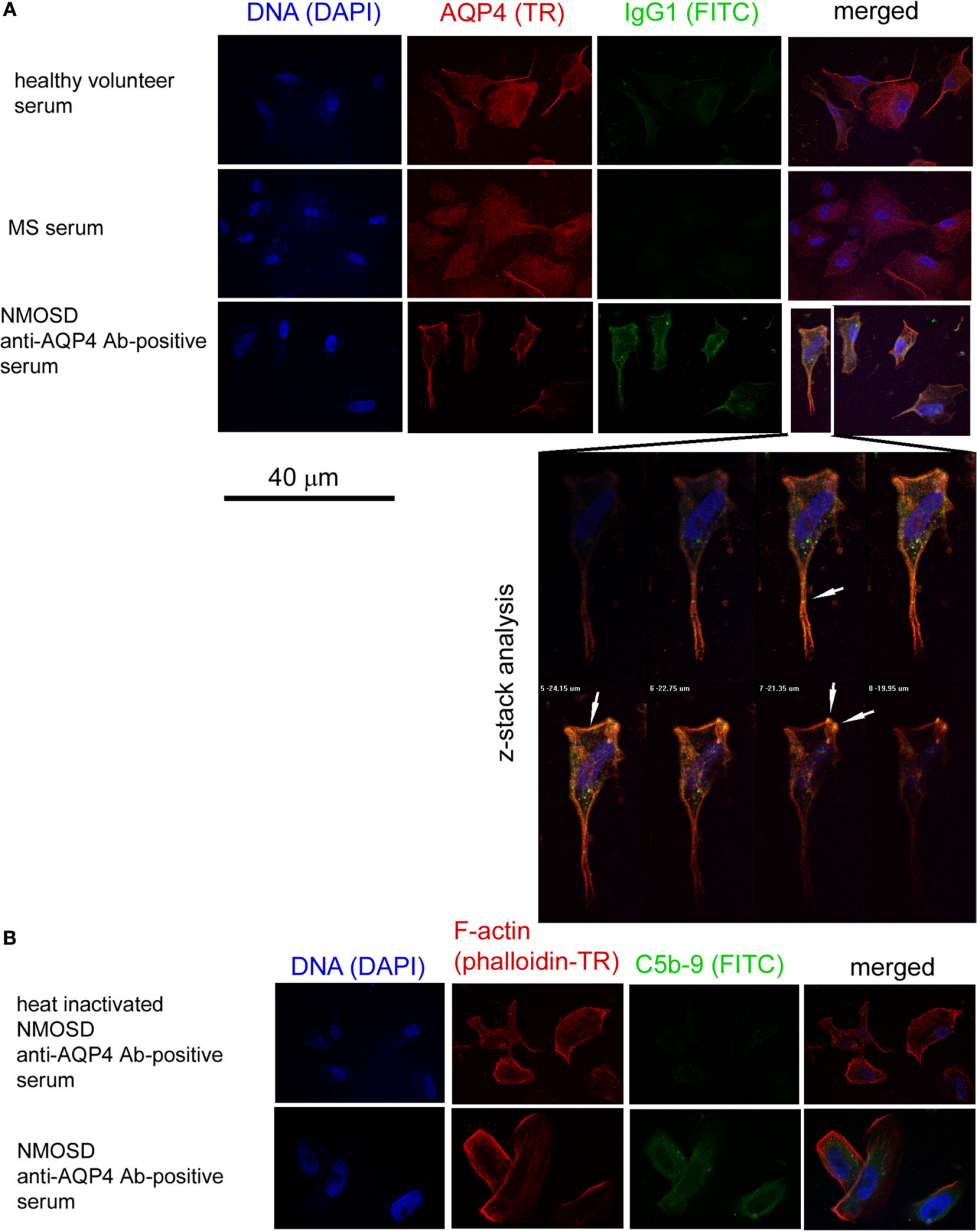
Anti-aquaporin-4 (AQP4) Ab-positive serum from neuromyelitis optica spectrum disorder (NMOSD) patients activates membrane attack complex deposition on the surface of human astrocytes. **(A)** Anti-AQP4 Ab-positive serum from NMOSD patients opsonizes human primary astrocytes. Confocal z-stack microscopy analysis confirms formation of AQP4–IgG1 immunological complexes on the cell surface in astrocytes incubated with NMOSD serum. White arrows mark colocalization of AQP4 and IgG1 signals (yellow color) **(B)**. Binding of AQP4 receptors by autoantibodies activates classical complement cascade which can destabilize astrocytes by formation of membrane attack complex (C5b-9) [**(B)**, lower panel]. Negative signal in NMOSD serum which has been previously heated causing complement inactivation (56°C, 30 min) excluded false positivity in C5b-9 ICC analysis [**(B)**, upper panel].

In the next set of experiments, we verified the pathogenic character of anti-AQP-4 Abs present in NMOSD serum toward astrocytes. As human astrocytes present in BBB acquire membrane proteins CD46 and CD55 that inhibit activation of the complement cascade (C_hu_) in contact with endothelial cells (ECs), we used astrocytes derived from human induced neural stem cells which had no contact with ECs, therefore did not express membrane complement inhibitory proteins (8). As IgG1 class immunoglobulins possess the strongest properties to trigger C_hu_ after binding the antigen, we used anti-IgG1 and anti-AQP4 serotype of IgG2, which does not cross-react with IgG1 mAbs, to detect the reactivity of anti-AQP4 Ab-positive serum against astrocytes. First, we demonstrated the formation of immunological complexes on the surface of astrocytes in a double-staining immunocytochemical analysis after 1-h incubation with NMOSD serum (10% v/v), contrary to RRMS or HC serum (Figure 2A). Confocal z-stack microscopy analysis confirmed the formation of IgG1–AQP4 complexes on the astrocyte surface in the presence of NMOSD serum (Figure 2A). Next, using the same incubation conditions, we detected C5b-9 formation (Figure 2B, lower panel). Negative signal obtained with heat-inactivated serum (56°C, 30 min), contrary to positive signal in non-heated serum confirmed that C5b-9 formation was an effect of NMOSD serum (Figure 2B).

### Formation of C5b-9 Complex Is Associated With Astrocyte Lysis and Decreased GFAP Expression Only in a Small Number of Cells

**Figure 3:**
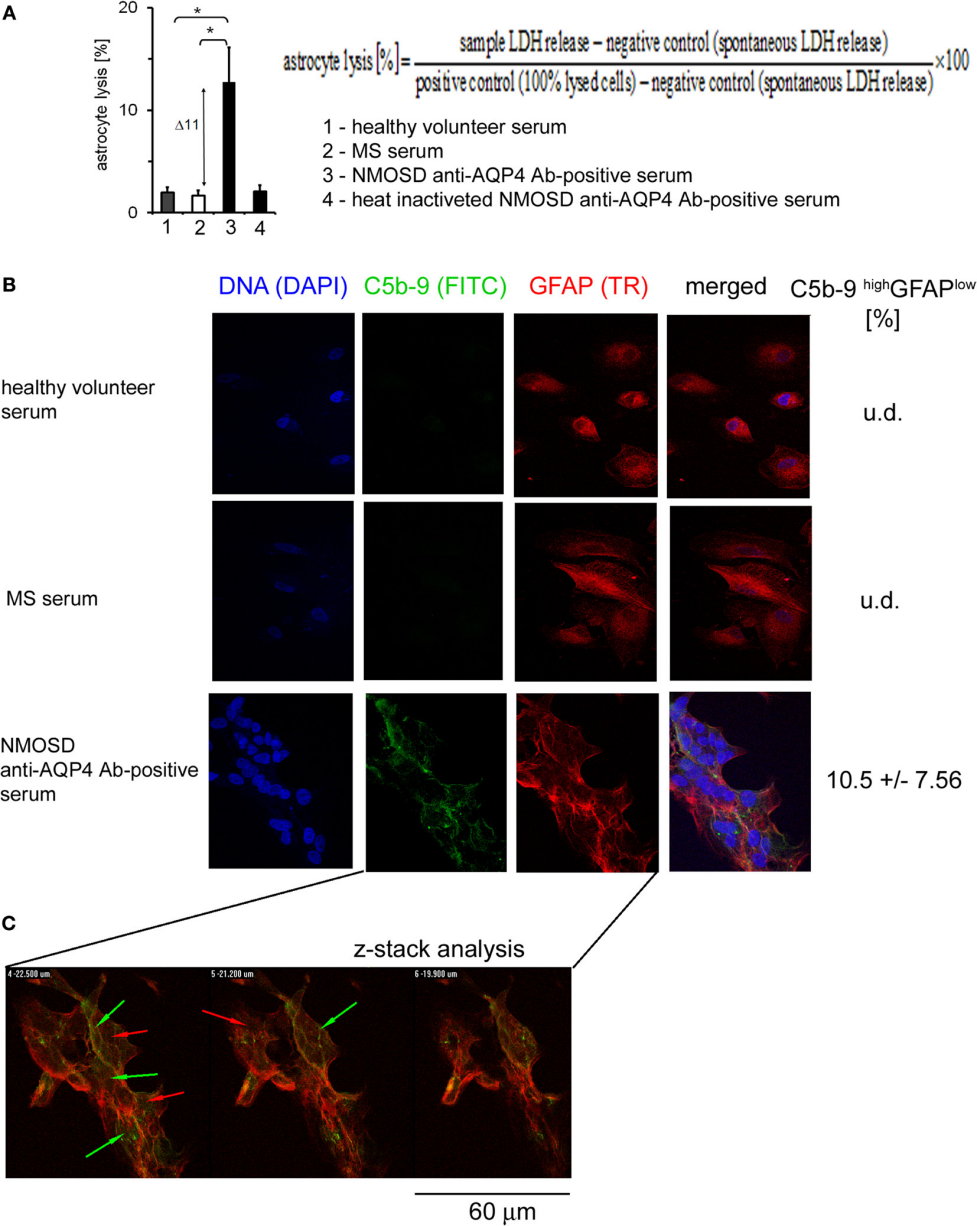
Formation of C5b-9 complexes is associated with cell lysis and glial fibrillary acidic protein (GFAP) expression loss by a little number of astrocytes. **(A)** 11% of astrocytes is lysed in the presence of neuromyelitis optica spectrum disorder (NMOSD) anti-aquaporin-4 (AQP4) Ab-positive serum (left panel). Cell lysis expressed as a percentage of the change of lactate dehydrogenase (LDH) concentration normalized to positive and negative controls according to formula presented in [**(A)**, upper right panel]. **(B)** Double-staining of astrocytes with C5b-9 and GFAP. Incubation of astrocytes with anti-AQP4 Ab-positive serum from NMOSD patients, opposite to multiple sclerosis (MS) and healthy control, activates C5b-9 complex formation (green arrows) accompanied by reduced GFAP expression (red arrows). **(C)** Confocal microscopy analysis confirms C5b-9 complex formation on the cell surface of astrocytes which characterized GFAP low expression.

In response to Ab-complement-mediated attack, cells can undergo lysis (>5% of lysed cells) or, when exposed to sublytic (<5% of lysed cells) amounts of C5b-9, become activated (25). To ensure how anti-AQP4 Ab-positive serum affects astrocytes, LDH was measured in the supernatants. We found that astrocytes exposed to anti-AQP4 Ab-positive serum, contrary to RRMS or HC serum, released increased amounts of LDH. Normalization of sample concentration to LDH levels present in positive and negative controls revealed that approximately 11% of astrocytes exposed anti-AQP4 Ab-positive serum underwent lysis (Figure 3A). This effect was dependent on the complement system as serum heating caused complement inactivation and reduction of LDH release.

Glial fibrillary acidic protein plays a central role in astrocyte–neuron interactions, responsible for the cytoskeleton structure of glia cells and for maintaining their mechanical strength, as well as supporting neighboring neurons and the BBB (26). Therefore, we investigated whether anti-AQP4 Ab-complement-induced attack can interfere with astrocyte GFAP expression. Double staining of astrocytes with C5b-9 and GFAP revealed that NMOSD serum, contrary to RRMS or HC serum, activated C5b-9 complex formation and reduced GFAP expression (Figure 3B). The z-stack confocal analysis confirmed C5b-9 complex formation on astrocyte cell surface in the presence of anti-AQP4 Ab-positive serum (Figure 3C). The fluorescence intensity analysis showed that only cells with high deposition of C5b-9 (high signal in ICC analysis) had decreased expression of GFAP (10% of all astrocytes), whereas the rest of astrocytes were characterized by low C5b-9 deposition and normal GFAP expression, comparable to astrocytes incubated with HC or RRMS serum (Figure 3C, green/red arrows). These findings showed that the majority astrocytes are resistant to AQP4-C_hu_-induced destruction, probably due to their membrane expression of complement inhibitory protein CD59 (8, 27), or sublytic C5b-9 concentration (28), suggesting that another parallel mechanism of astrocyte destruction in NMOSD inflammation should exist.

### Astrocytes Recruit Neutrophils in the Presence of Anti-AQP-4 Ab NMOSD Serum as a Result of C5a Release

**Figure 4:**
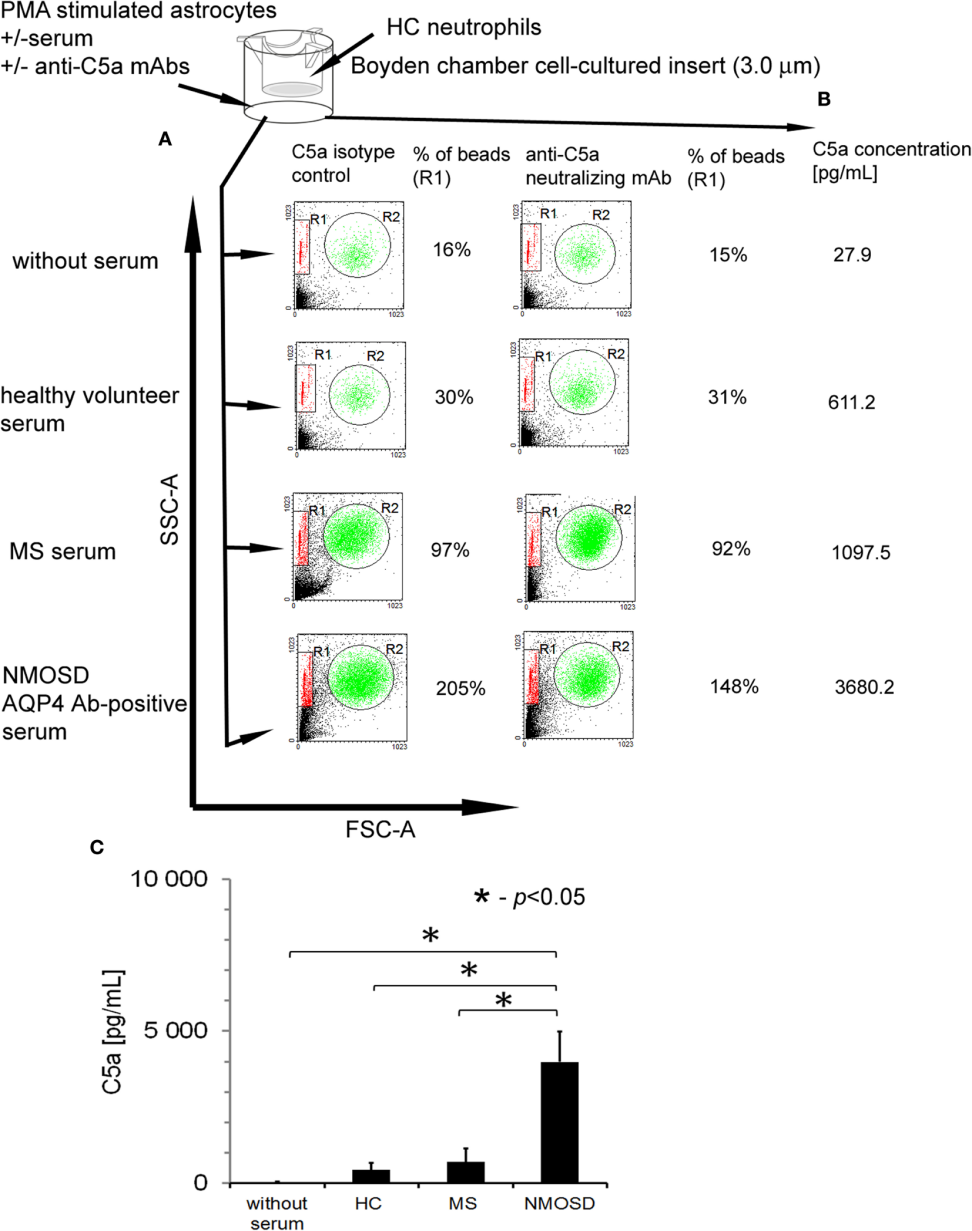
Human astrocytes recruit healthy control (HC) neutrophils in the presence of anti-aquaporin-4 (AQP4) Ab-positive neuromyelitis optica spectrum disorder (NMOSD) serum as a result of C5a release. **(A)** Astrocytes preincubated with NMOSD serum induce chemotaxis of HC neutrophils as the result of C5a release. The example of analysis representative of four independent experiments. R1—region of beads; R2—region of neutrophils. **(B)** C5a release following AQP4–IgG1 binding to AQP4 during incubation of primary human astrocytes with NMOSD serum. Concentration of C5a was measured in supernatants after 1 h incubation with NMOSD, remitting–relapsing multiple sclerosis, or HC serum. **(C)** Concentration of C5a in supernatants of astrocytes incubated with NMOSD or control serum. The bars represent the means ± SD from four independent experiments.

To confirm that anti-AQP-4 Ab binding astrocytes induce inflammation *via* recruitment of neutrophils, we examined chemotaxis capacity of HC neutrophils in the presence of astrocytes preincubated with 10% NMOSD serum. Serum without astrocytes and astrocytes stimulated by PMA in the absence of serum were used as the controls. We found that NMOSD and RRMS sera alone possessed weak chemotaxis properties toward neutrophils (9% of beads). Moreover, PMA-stimulated astrocytes in the absence of serum possessed chemotaxis property (16% of beads), probably as the result of the release of CXCL1 (29, 30) (Figure 4A). HC neutrophil chemotaxis was potently increased in the presence of astrocytes preincubated with NMOSD serum, compared with RRMS or HC serum (205% of beads in NMOSD, 97% in RRMS, and 30% in HC). When neutralizing anti-C5a mAbs were used, inhibition of HC neutrophil chemotaxis about 57% was detected only in NMOSD serum incubations, in oppose to RRMS serum (Figure 4A, right panel). This was accompanied by the high concentration of C5a detected in supernatants during incubation of astrocytes with NMOSD serum, unlike RRMS or HC serum (Figures 4B,C).

### Preactivated HC Neutrophils Polarize Astrocytes to Proinflammatory Cells and Enhance Glutamate Pump Dysregulation

**Figure 5:**
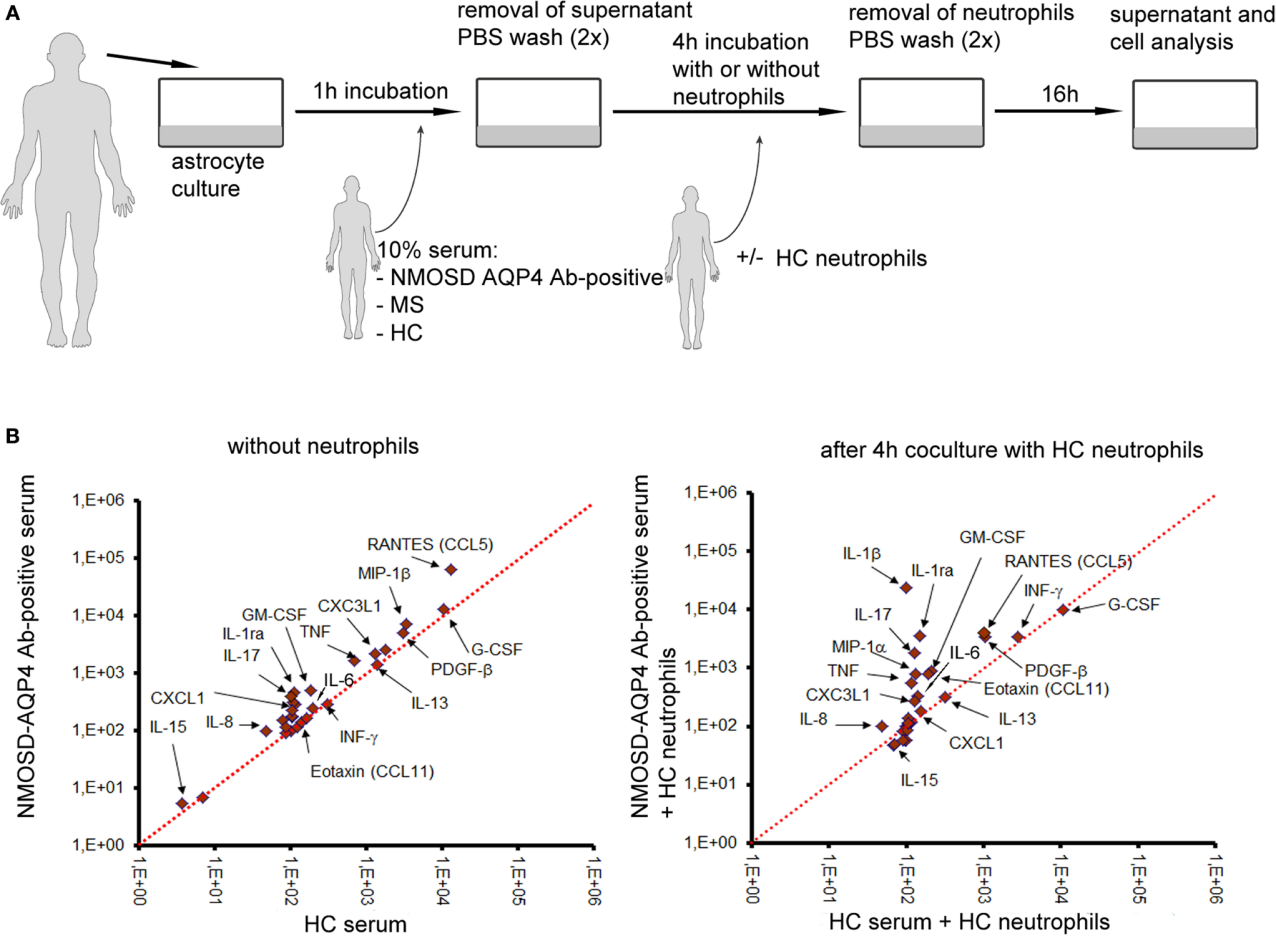

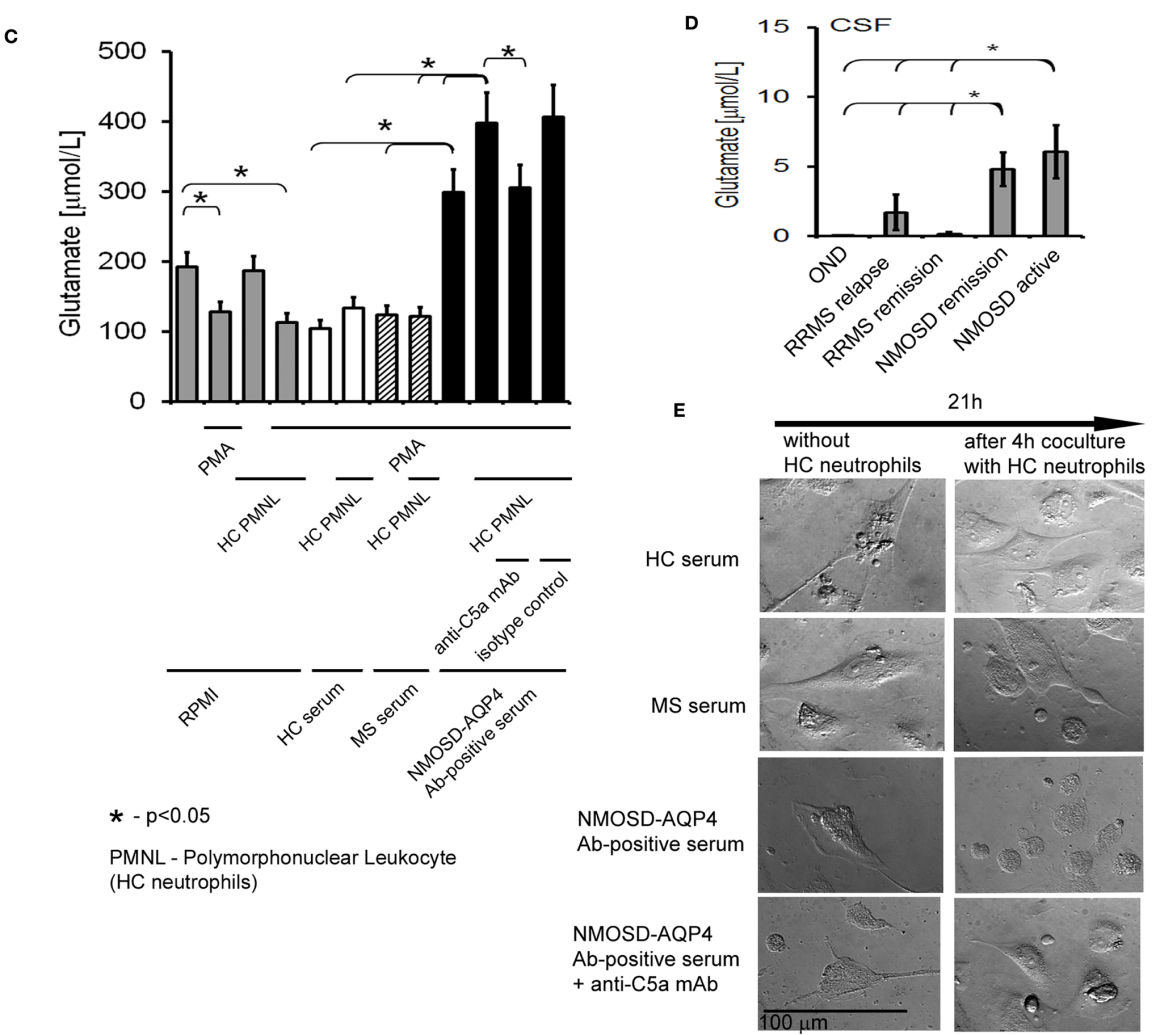
Preactivated healthy control (HC) neutrophils polarize astrocytes into proinflammatory cells and enhance glutamate pump dysregulation. **(A)** An *in vitro* model of neuromyelitis optica spectrum disorder (NMOSD) inflammation consisting of NMOSD serum, human astrocytes, and HC neutrophils. As controls, multiple sclerosis (MS) serum, HC serum, and astrocytes without serum were used. **(B)** Human astrocytes produce proinflammatory cytokines and chemokines after incubation with NMOSD serum (left panel), and anti-aquaporin-4 (AQP4) Ab binding astrocytes trigger C5a-induced neutrophil recruitment which subsequently enhances proinflammatory cytokine production by astrocytes (right panel). **(C)** Anti-AQP4 Ab-positive serum in cooperation with HC neutrophils destabilizes astrocytes and reverses glutamate pump resulting in high glutamate concentration in extracellular space. **(D)** Cerebrospinal fluid (CSF) from NMOSD patients both in the active and remission stages contains higher concentration of glutamate, compared with remitting–relapsing multiple sclerosis (RRMS) or other neurological disorders (OND). **(E)** Anti-AQP4 Ab-positive serum in cooperation with HC neutrophils interrupts active transport of H_2_O by aquaporin-4 (AQP4) channel in astrocytes. DIC microscopy analysis reveals reduction of astrocyte volume and disruption of their shape. Images were acquired using Zeiss Axiovert 200 inverse microscope with a Zeiss Plan-apochromat 63×/1.43 differential interference contrast objective after 21 h incubation.

The last question we addressed was how antibody-complement–neutrophil axis affects function of astrocytes and what is the role of neutrophils in this process? We assumed that in NMOSD, astrocytes bind anti-AQP4 Abs which activate complement cascade which in turn induces neutrophil recruitment and preactivation *via* C5a. As only a subset of astrocytes was destructed as a consequence of C5b-9 formation, we hypothesized that preactivated neutrophils might deregulate the remaining astrocytes. Therefore, we created an *in vitro* model of NMOSD inflammation, in which human astrocytes were first exposed to anti-AQP4 Ab-positive NMOSD serum and subsequently incubated with HC neutrophils (Figure 5A). In this set of experiments, we used NMOSD serum collected during active phase of the disease, not only as anti-AQP4 Abs are typically not produced intrathecally resulting in their low concentration in CSF (31) but also because serum contains all complement components needed for the reaction. Supernatants collected at three time points were analyzed for 27 pro- and anti-inflammatory cytokines and chemokines by multi ELISA, as well as for glutamate and C5a concentrations. In addition, the morphology of astrocytes was analyzed using differential interference contrast microscopy.

We found that astrocytes exposed to anti-AQP4 Ab-positive NMOSD serum produced increased amounts of proinflammatory cytokines TNF, GM-CSF, IL-17, and IL-1β and chemokines CXCL1, IL-8, and RANTES in opposition to incubations with HC serum (Figure 5B; Table S1 in Supplementary Material) or RRMS serum (data not shown). Subsequent incubation of astrocytes with HC neutrophils further enhanced proinflammatory cytokine and chemokine production, making the differences between astrocytes exposed to NMOSD serum and control groups more clearly visible (Figure 5B; Table S1 in Supplementary Material). High levels of INF-γ, IL-8, MIP-1β, IL1b, and MIP-1α production by astrocytes exposed to NMOSD serum and HC neutrophils corresponded with their high concentrations in NMOSD CSF in the active stage of disease. In the same way, low IL-10 production corresponded with low concentration in the active NMOSD CSF.

Dysregulation of extracellular glutamate is another possible mechanism of NMOSD pathogenesis. Astrocytes are the major cell types that regulate extracellular glutamate concentration in brain, by downregulating it when it is briefly increased at glutamatergic synapse and maintaining low extracellular levels at rest (4). The analysis of glutamate concentration in supernatants revealed its increased level only in the incubation in which astrocytes were first exposed to NMOSD serum (Figure 5C). In the presence of HC neutrophils, glutamate concentration was further increased, while neutralizing anti-C5a mAbs reversed this effect. Glutamate level was not affected in other experimental settings, for example, astrocytes exposed to HC or RRMS serum, astrocytes exposed to HC or RRMS serum and subsequently to HC neutrophils. *In vivo* analysis of glutamate in CSF confirmed its high concentration in NMOSD patients in comparison to other studied groups (Figure 5D). The reverse of glutamate pump out into extracellular space might be a result of disruption of AQP4 receptors and, as a consequence, active transport of H_2_O to extracellular space. Low levels of H_2_O in cells result in decreasing cell volume. The living cell analysis by DIC microscopy revealed the reduction of cell volume and disruption of astrocyte shape only in the incubations in which astrocytes first were exposed to NMOSD serum and subsequently to HC neutrophils (Figure 5E, low-right panel). Neutralizing anti-C5a mAbs reversed this effect. In other studied incubations, no changes in astrocyte morphology were observed (Figure 5E).

## Discussion

Although many questions were raised about the autoimmune pathogenesis of NMOSD, the role of neutrophils remains unsolved. As histological findings demonstrated complement deposits associated with granulocyte infiltration in postmortem NMOSD brains (5, 7, 19), it has been suggested that pathological engagement of neutrophils in NMOSD might be an effect of complement system activation (6). Here, we demonstrated the key role of complement activation triggered by AQP4–IgG binding AQP4 on astrocytes in exclusive recruitment and activation of neutrophils in NMOSD. This reaction determines the release of the anaphylatoxin C5a which is a specific chemoattractant for neutrophils that contrary to T, B, or NK cells, possess high expression of C5a receptors (CD88) (32, 33). Previous studies assessing chemokines and cytokines related to neutrophil recruitment in NMOSD demonstrated increased levels of IL-8 and IL-17 in CSF (34) as well as ENA-78, CCL4, or G-CSF in serum (17, 35). We found that among chemotaxis factors responsible for neutrophil or eosinophil recruitment, only IL-8 level was elevated in NMOSD CSF. Conversely, we demonstrated significantly higher concentration of C5a in NMOSD CSF in comparison to RRMS or OND CSF which was reflected by increased C5a concentration in NMOSD serum. Increased concentrations of ENA-78, CCL4, and G-CSF found in the serum of NMOSD patients may be a result of their release by preactivated circulating neutrophils (36). The differences between the studies may be due to the variations in time of sample collection and inclusion of NMOSD patients undergoing immunosuppressive/immunomodulatory therapies. In this study, samples collected in acute phase of the disease were obtained from treatment-naïve patients.

Next, we demonstrated the vital role of C5a for preactivation of HC neutrophils *in vitro* and *ex vivo* in NMOSD patients. Experiments, in which HC neutrophils were preincubated with NMOSD CSF in the presence of neutralizing anti-C5a mAbs, revealed that C5a was responsible for increased neutrophil chemotaxis, diapedesis, adhesion molecule expression, phagocytosis, and ROI production. These *in vitro* experiments correlated with *ex vivo* findings demonstrating preactivation of peripheral blood neutrophils in NMOSD patients. Neutrophils in NMOSD were not fully activated, as neutrophils should receive two stimulating signals, or directly recognize a pathogen *via* toll-like receptors. In NMOSD, inflammation is aseptic; therefore, neutrophils received only one signal from the site of inflammation. Preactivation is a sensitive process which is mediated when neutrophils receive and recognize stimulus present at a very low concentration, which is dependent on the intensity of immunological reaction and distance of circulating neutrophils from the site of inflammation. In fact, C5a is able to preactivate neutrophils at a concentration of 1 pg/mL which results in intracellular calcium mobilization, stimulation of chemotaxis, aggregation, but not activation (37). We found very high concentration of C5a in NMOSD CSF (>4,000 pg/mL) and low concentration in NMOSD serum (43.5 ± 13.98 pg/mL). This explains why peripheral blood neutrophils are pre-activated but not fully activated and why they migrate to CNS. The key role of complement activation was also supported by a recent pilot study showing a beneficial effect of eculizumab, a humanized monoclonal IgG that neutralizes C5 and its cleavage to C5a, in the treatment of active NMOSD (38).

Physiological regulation of complement activation is dependent on the concentration of autoantibody, class of immunoglobulin, and presence of complement protection proteins on the cell surface (39). Therefore, for subsequent experiments patients’ sera collected during the active phase of the disease, which contained all complement components and autoantibodies at concentrations adequate to clinical symptoms, were used. We have demonstrated that anti-AQP4 Ab-positive NMOSD serum activated complement cascade which resulted in C5a release and C5b-9 complexes formation, astrocyte lysis, and loss of GFAP expression. In addition, we provided evidence that C5a released during this reaction was functionally active, as supernatant collected after incubation of astrocytes with NMOSD serum was able to preactivate and recruit HC neutrophils. ICC analysis showed colocalization of IgG1 with AQP4 protein, as well as GFAP downregulation associated with C5b-9 formation, confirming the crucial role of anti-AQP4 Ab binding triggering complement cascade. However, only a small subset (~10–11%) of cultured astrocytes was lysed and demonstrated GFAP loss as a result of C5b-9 formation, even though all astrocytes were opsonized by IgG1. This might be a result of membrane expression of complement regulatory proteins on astrocyte surface (27, 28) or sublytic concentration of C5b-9 (28). Postmortem histological analysis revealed that in normal human brain, opposite to other organs, astrocytes are devoid of CD46 (membrane cofactor protein), CD55 (decay accelerating factor), or CD59 (protectin) that inhibit activation of the C_hu_ cascade (8). However, some of *in vitro* studies demonstrated these proteins in cultured human astrocytes (28, 39–41). One of the recent studies showed that human brain cortical astrocytes are negative for membrane CD46 and CD55, but positive for membrane CD59 (8). Based on the results of our study, it is highly probable that astrocytes used in our experiments possessed membrane complement inhibitory proteins, as the majority of astrocytes retained adherence and produced increased amounts of chemokines/cytokines. Consequently, we concluded that there should be another mechanism by which astrocytes are pathologically affected. Therefore, we examined the role of preactivated neutrophils in astrocyte damage. Previous studies have highlighted the role of glutamate, which high concentration, as the result of disruption of AQP4 channel, is cytopathic for neurons (42). To address this issue, we constructed an *in vitro* model of NMOSD inflammation, in attempt to visualize how anti-AQP4 Abs, complement system and neutrophils affect together astrocyte status. Primary cultured human astrocytes were first exposed to NMOSD anti-AQP4 Ab-positive serum and subsequently to freshly isolated neutrophils from HCs. By using HC instead of NMOSD neutrophils, we tried to mimic all the pathophysiological mechanisms responsible for transition from health to pathology. This was not possible with peripheral blood neutrophils from NMOSD as they were already preactivated during the course of the disease (16). Glutamate concentration in supernatants was the highest in the experiments in which astrocytes were first exposed to anti-AQP4 Ab-positive serum, and subsequently to HC neutrophils, suggesting that neutrophils are the essential element to complete pathological reaction. Two mechanisms could be responsible for pathological glutamate transport following anti-AQP4 Ab binding. First one might be dependent on excitatory aminoacid transporter 2 (EAAT2) downregulation which affects glutamate uptake (43). Another possibility is that glutamate transport by the plasma membrane transporters is reversible and depends on [Na^+^]/[K^+^] extra/intracellular concentration. Therefore, anti-AQP4 Abs interfere with Na^+^–K^+^ ATPase and K^+^ channels affecting [Na^+^]/[K^+^] extra and intracellular levels. As a consequence, when intracellular [Na^+^]/extracellular [K^+^] increases or extracellular [Na^+^]/intracellular [K^+^] decreases, glutamate can be transported in the outward direction (44). This relationship is a result of clustering AQP4 water channel with other proteins, Na^+^, K^+^-ATPaze, K^+^ channels, EAAT2, and mGluR5 present in astrocyte membrane (45). We also demonstrated that astrocytes reduced their cell volume and changed their shape. The disruption of AQP4 receptors resulted in inhibition of active transport of H_2_O to intracellular space and decrease in cell volume, which was additionally facilitated by proinflammatory environment mediated by preactivated neutrophils. This set of experiments revealed that astrocyte destabilization and dysregulation of glutamate pump require cooperation of anti-AQP4 Abs and neutrophils.

We also showed an unexpected role of C5a-preactivated neutrophils in induction of reactive astrocytes producing TNF, GM-CSF, IL-1, and IL-17 and chemokines CXCL1, IL-8, and RANTES. Interestingly, this effect was not observed when astrocytes were exposed to freshly isolated HC neutrophils, or HC neutrophils incubated with HC serum. The increased cytokine production by astrocytes corresponded with high cytokine concentrations in CSF, providing a link between *in vitro* findings and pathophysiology of NMOSD *in vivo*. In BBB model, astrocytes cocultured with epithelial cells and exposed to NMO–IgG produced increased amounts of IL-6, which induced CCL2 and IL-8 chemokine production by adjacent microvascular ECs (46). These data partially corresponded with our findings showing increased IL-8 levels both in NMOSD CSF and in supernatants after incubation of astrocytes with NMOSD serum and HC neutrophils, while IL-6 level was not significantly increased in NMOSD CSF, however, was elevated in astrocyte culture supernatants. Reactive astrocytes together with preactivated neutrophils create proinflammatory environment which might influence the status of other cells in the CNS: neurons, oligodendrocytes, and brain-infiltrating T lymphocytes (46, 47).

A limitation of our study is a small number of participants in each group, and nearly equal gender distribution in NMOSD group, which is not a representative epidemiological distribution for MNOSD, where male to female ratio is about 1:3.6 to 1:10.4 (1). However, no data are available on the major differences in neutrophil function, complement component concentration and astrocyte morphology depending on the patient gender. Therefore, the results presented in this study seem to be relevant to both genders.

**Figure 6:**
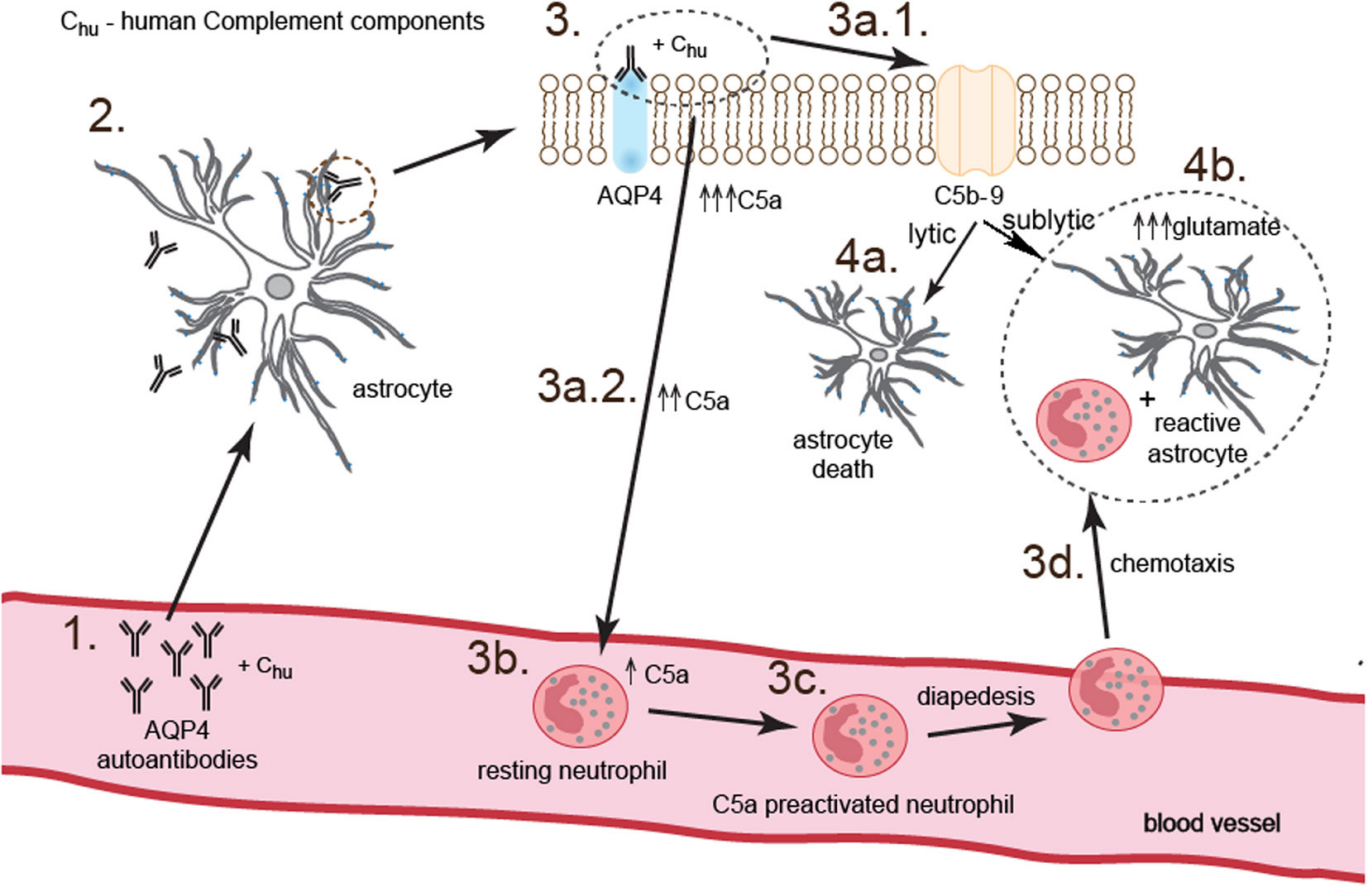
Anti-aquaporin-4 (AQP4) Ab binding astrocytes initiate two parallel complementary reactions. The first one is dependent on the complement cytotoxicity *via* C5b-9 complex formation (pathway 3.–3a.1.–4a.), and the second one on the sublytic effect of C5b-9 and dysregulation of astrocyte glutamate pump into extracellular space by C5a-preactivated neutrophils (pathway 3.–3a.2.–3b.–3c.–3d.–4b.). As a consequence, astrocytes are partially destroyed; however, a major population of astrocytes polarizes into proinflammatory cells which produce cytokines and chemokines and are characterized by pathological glutamate removal from extracellular space.

To summarize, our study demonstrated that NMOSD pathogenesis is based on the two parallel processes triggered by binding anti-AQP4 Abs to AQP4 on the surface of astrocytes. First phase is dependent on complement system activation and neutrophil recruitment and preactivation *via* C5a. This phase is accompanied by physical destruction of a small rate of astrocytes (10%) by C5b-9 complex. Second phase is dependent on the C5a-preactivated neutrophils which, by providing proinflammatory environment, mediate reactive astrocyte formation and facilitate impairment of astrocyte function *via* toxic effect of glutamate (Figure 6).

## Ethics Statement

The study was approved by the Ethics Committee of the Medical University of Lodz (RNN/44/14/KE), and informed consent was obtained from each participant of the study.

## Author Contributions

PPiatek, MD, and PL designed research; PPiatek, MD, PPrzygodzka, KD, MM, and PL performed research; PL contributed new reagents/analytic tools; NL and PL analyzed data and wrote the paper.

## Conflict of Interest Statement

The authors declare that the research was conducted in the absence of any commercial or financial relationships that could be construed as a potential conflict of interest.

## Supplementary Material

Figure S1 Analysis of cell morphology, purity, and viability. **(A)** Phenotypic analysis of purified neutrophils. Flow cytometry analysis of CD14 (clone M5E2, BD), CD15 (MMA, BD), CD16 (3G8, BD), and 7-AAD in the population of neutrophils isolated by Polymorhprep™ gradient separation was performed using flow cytometry (LSRII, BD) and BD FACSDiva™ analysis software. Cells with CD15^high^/7-AAD^−^ expression were identified as live neutrophils (~97%), cells with CD14^high^CD15^low^ expression as monocytes (1.4%), and cells with CD16^−^CD15^+^ (less than 0.5%) expression as eosinophils. **(B)** ICC analysis of human matured astrocytes based on the glial fibrillary acidic protein (GFAP) expression (red pseudocolor). Astrocytes were derived from human induced neural stem cells.

Figure S2 Expression of CD11b, CD18, and CD62L adhesion molecules responsible for diapedesis of neutrophils to cerebrospinal fluid (CSF) in neuromyelitis optica spectrum disorder (NMOSD), remitting–relapsing multiple sclerosis (RRMS), and other neurological disorders (OND) patients. **(A)** NMOSD CSF affects healthy control (HC) neutrophil adhesion molecule expression *via* C5a, contrary to RRMS or OND. The bars represent the mean expression (MFI) ± SD from four independent experiments. **(B)** *Ex vivo* analysis of adhesion molecule expression on peripheral blood neutrophils in NMOSD, RRMS, and OND patients and HC reveals higher expression of CD11b, CD18, and CD62L in the active NMOSD, contrary to the other studied groups. Data are presented as the means of MFI ± SD.

Figure S3 Phagocytosis of neutrophils in neuromyelitis optica spectrum disorder (NMOSD), remitting–relapsing multiple sclerosis (RRMS), and other neurological disorders (OND) patients. **(A)** Cerebrospinal fluid (CSF) derived from NMOSD patients in the active stage primes healthy control (HC) neutrophils for more intensive *E. coli* phagocytosis, while CSF collected from RRMS patients in the active or remission stages or OND CSF has no effect on neutrophil phagocytosis. **(B)** Anti-C5a neutralizing Abs reverse NMOSD CSF effect on HC neutrophil phagocytosis. Histograms depict mean intensity of *E. coli* phagocytosis ± SD taken from four independent experiments. **(C)** *Ex vivo* analysis of peripheral blood neutrophil phagocytosis in NMOSD, RRMS, OND, and HC reveals higher phagocytic activity of NMOSD neutrophils in the active stage of disease, contrary to the other studied groups. Data are presented as the means ± SD.

Figure S4 Neutrophil reactive oxygen intermediate (ROI) production in neuromyelitis optica spectrum disorder (NMOSD), remitting–relapsing multiple sclerosis (RRMS), and other neurological disorders (OND) patients. **(A)** Cerebrospinal fluid (CSF) derived from NMOSD patients in the active and remission stages, and CSF from relapse RRMS primes healthy control (HC) neutrophils for more intensive ROI production after PMA stimulation in Bursttest analysis. **(B)** Contrary to RRMS, preactivation of HC neutrophils by NMOSD CSF is depended on C5a. Histograms depict mean intensity of ROI production ± SD taken from four independent experiments. **(C)** *Ex vivo* analysis of ROI production in circulating neutrophils in the patients with NMOSD, RRMS, OND, and HC reveals no differences in non-stimulated neutrophils, while following stimulation with PMA neutrophils from active NMOSD and relapse RRMS produce higher amounts of ROI. Data are presented as the means ± SD.

Table S1 C5a-preactivated healthy control (HC) neutrophils polarize astrocytes into proinflammatory cells. Statistically significant differences between (a) astrocytes vs. astrocytes + neuromyelitis optica spectrum disorder (NMOSD) serum and (b) astrocytes + NMOSD serum vs. astrocytes + NMOSD serum + HC neutrophils. Data are presented as the means ± SEM from four independent experiments.
