# Supplementary figures and images for: C5a-Preactivated Neutrophils Are Critical for Autoimmune-Induced Astrocyte Dysregulation in Neuromyelitis Optica Spectrum Disorder
Source: Front Immunol. 2018 Jul 23;9:1694. doi: 10.3389/fimmu.2018.01694 (PMC6065055; doi:10.3389/fimmu.2018.01694)

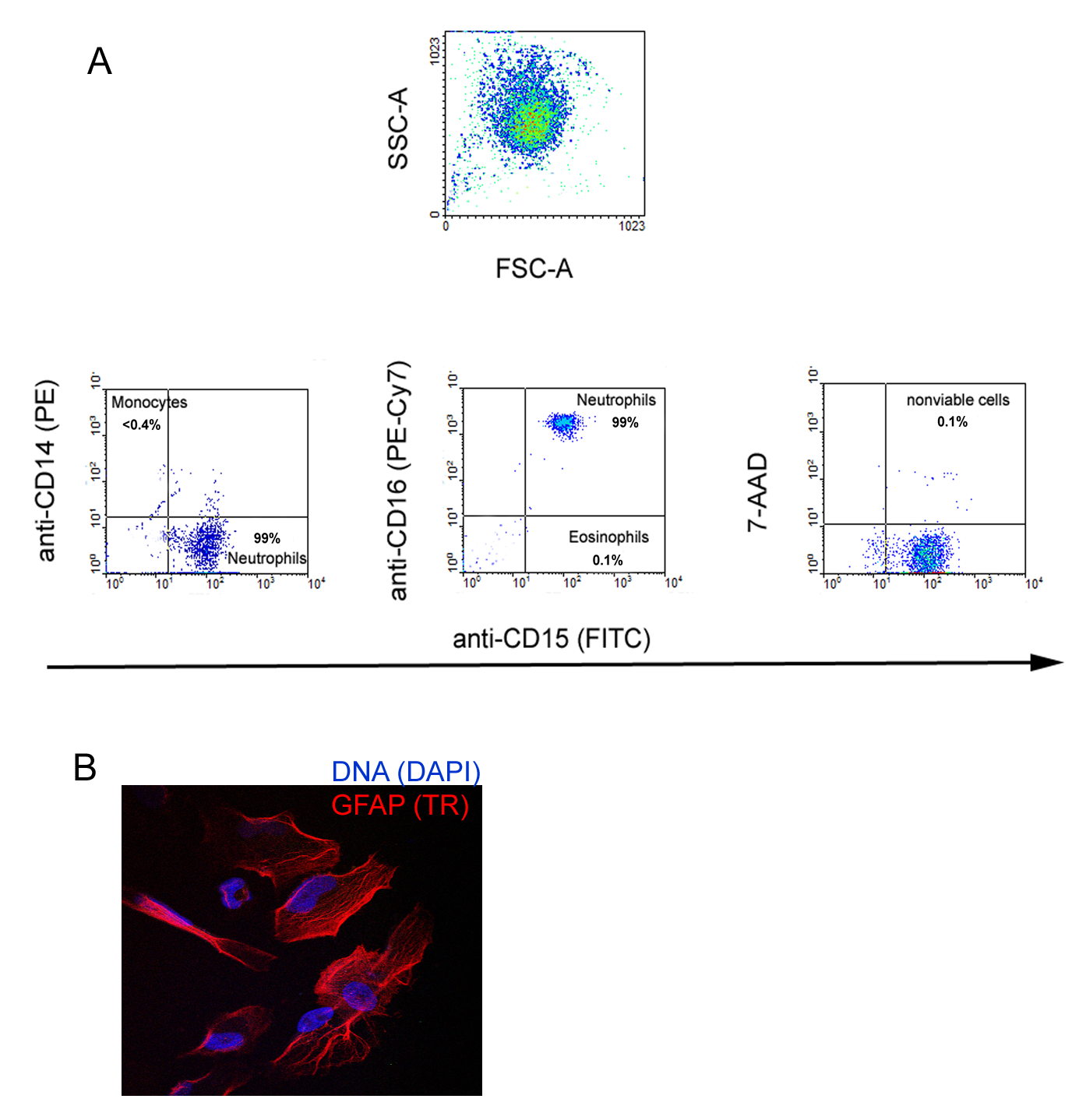

Supplement: Figure S1 — Analysis of cell morphology, purity, and viability. (A) Phenotypic analysis of purified neutrophils. Flow cytometry analysis of CD14 (clone M5E2, BD), CD15 (MMA, BD), CD16 (3G8, BD), and 7-AAD in the population of neutrophils isolated by Polymorhprep™ gradient separation was performed using flow cytometry (LSRII, BD) and BD FACSDiva™ analysis software. Cells with CD15high/7-AAD− expression were identified as live neutrophils (~97%), cells with CD14highCD15low expression as monocytes (1.4%), and cells with CD16−CD15+ (less than 0.5%) expression as eosinophils. (B) ICC analysis of human matured astrocytes based on the glial fibrillary acidic protein (GFAP) expression (red pseudocolor). Astrocytes were derived from human induced neural stem cells. [file image_1.tif]

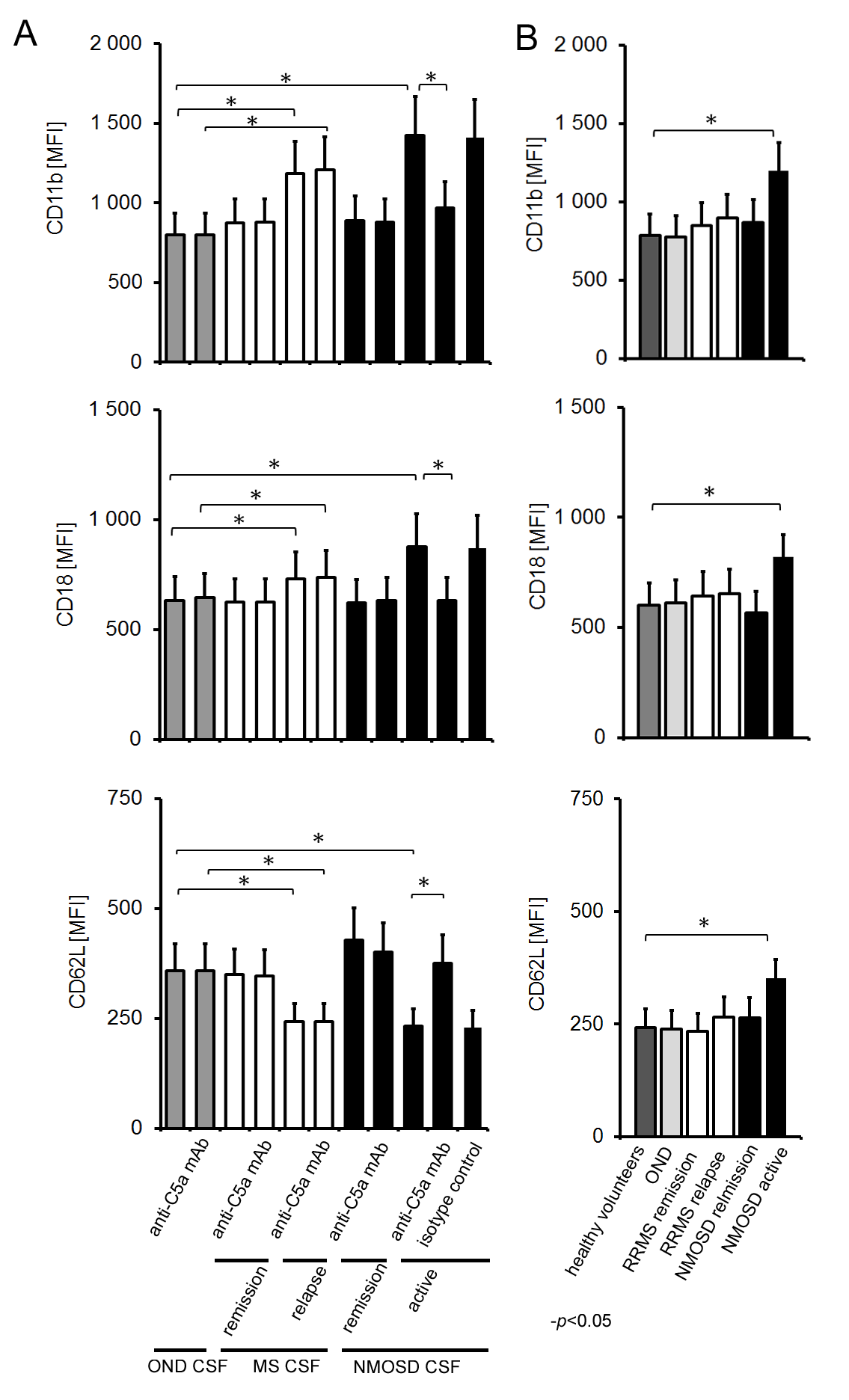

Supplement: Figure S2 — Expression of CD11b, CD18, and CD62L adhesion molecules responsible for diapedesis of neutrophils to cerebrospinal fluid (CSF) in neuromyelitis optica spectrum disorder (NMOSD), remitting–relapsing multiple sclerosis (RRMS), and other neurological disorders (OND) patients. (A) NMOSD CSF affects healthy control (HC) neutrophil adhesion molecule expression via C5a, contrary to RRMS or OND. The bars represent the mean expression (MFI) ± SD from four independent experiments. (B) Ex vivo analysis of adhesion molecule expression on peripheral blood neutrophils in NMOSD, RRMS, and OND patients and HC reveals higher expression of CD11b, CD18, and CD62L in the active NMOSD, contrary to the other studied groups. Data are presented as the means of MFI ± SD. [file image_2.tif]

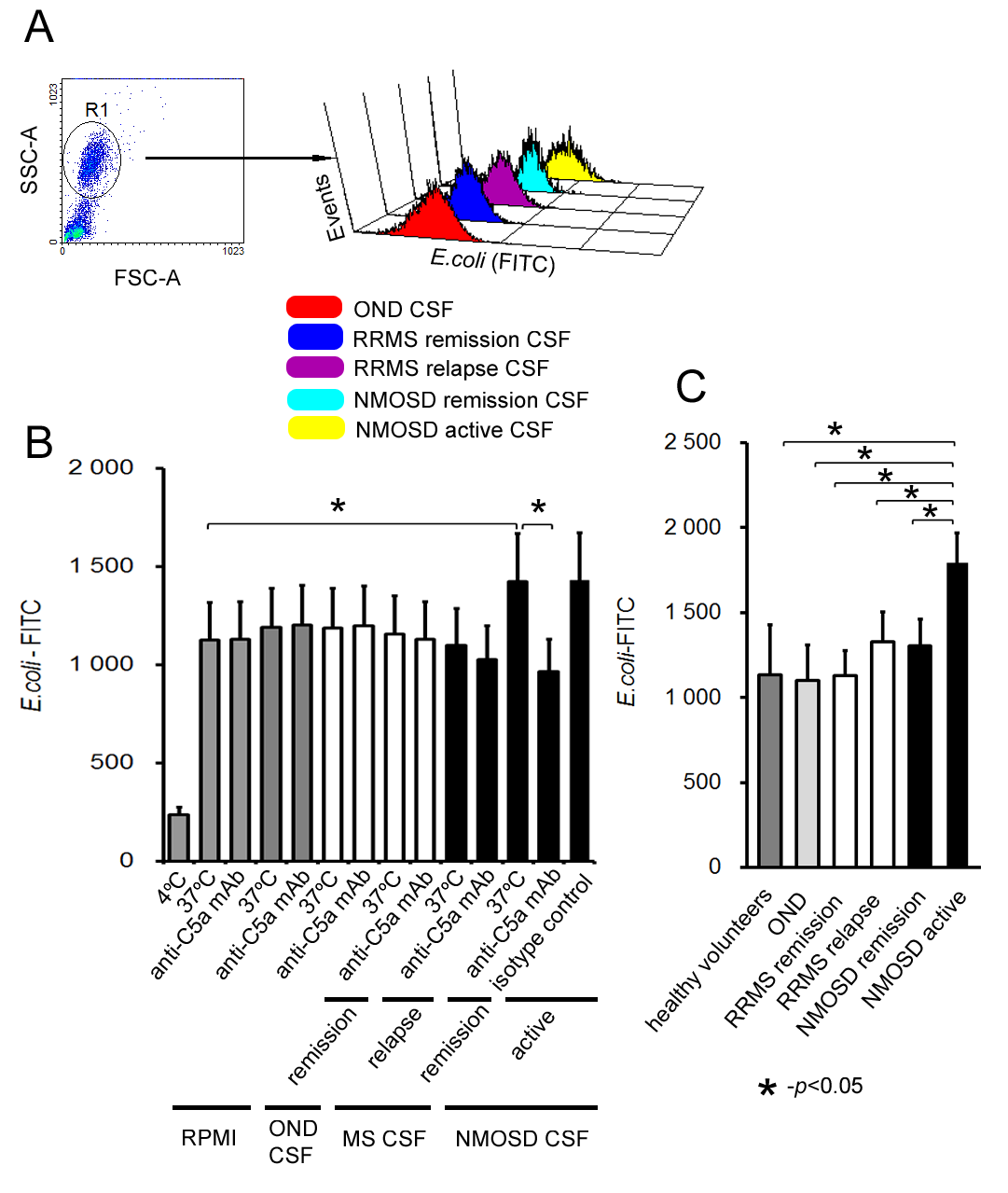

Supplement: Figure S3 — Phagocytosis of neutrophils in neuromyelitis optica spectrum disorder (NMOSD), remitting–relapsing multiple sclerosis (RRMS), and other neurological disorders (OND) patients. (A) Cerebrospinal fluid (CSF) derived from NMOSD patients in the active stage primes healthy control (HC) neutrophils for more intensive E. coli phagocytosis, while CSF collected from RRMS patients in the active or remission stages or OND CSF has no effect on neutrophil phagocytosis. (B) Anti-C5a neutralizing Abs reverse NMOSD CSF effect on HC neutrophil phagocytosis. Histograms depict mean intensity of E. coli phagocytosis ± SD taken from four independent experiments. (C) Ex vivo analysis of peripheral blood neutrophil phagocytosis in NMOSD, RRMS, OND, and HC reveals higher phagocytic activity of NMOSD neutrophils in the active stage of disease, contrary to the other studied groups. Data are presented as the means ± SD. [file image_3.tif]

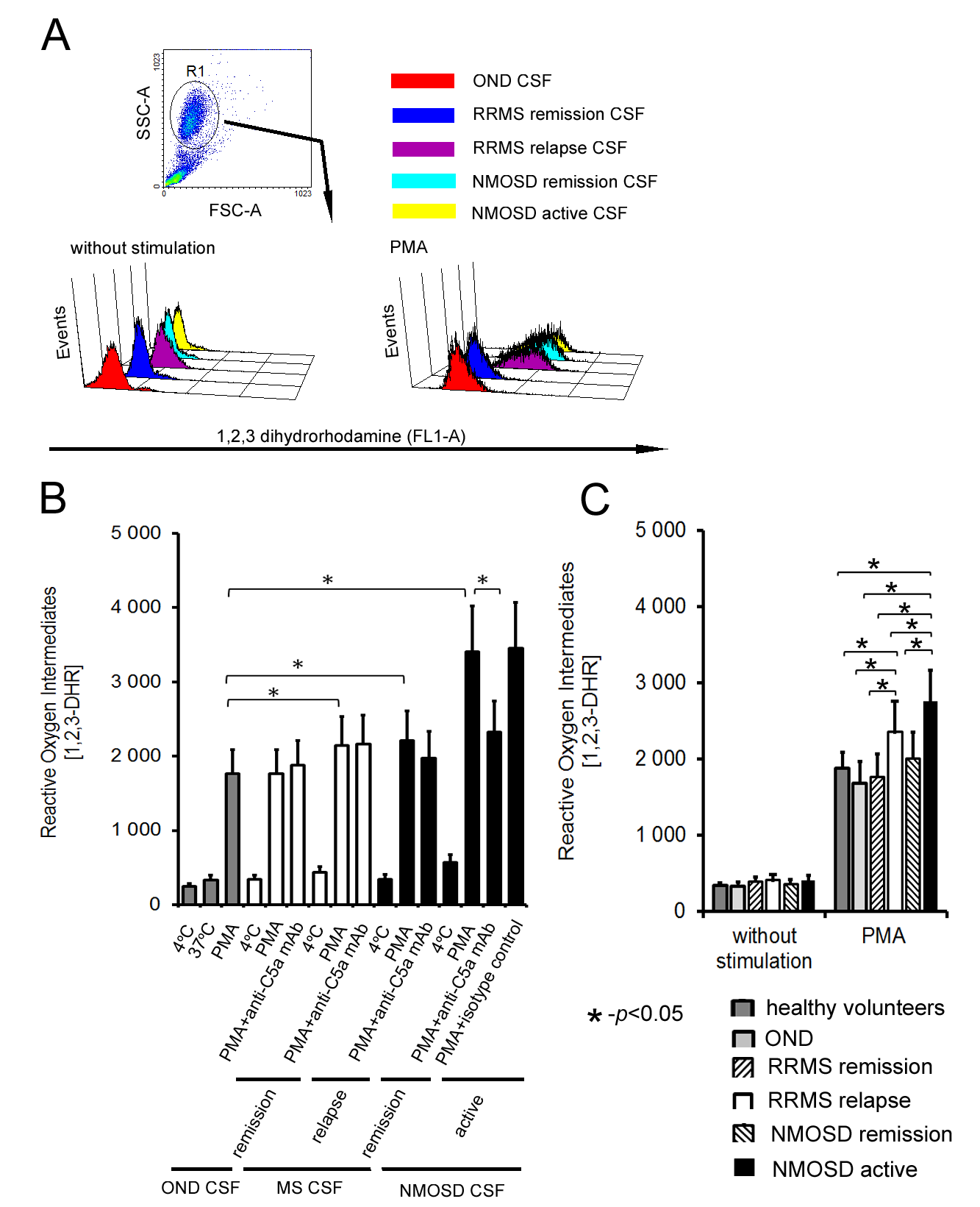

Supplement: Figure S4 — Neutrophil reactive oxygen intermediate (ROI) production in neuromyelitis optica spectrum disorder (NMOSD), remitting–relapsing multiple sclerosis (RRMS), and other neurological disorders (OND) patients. (A) Cerebrospinal fluid (CSF) derived from NMOSD patients in the active and remission stages, and CSF from relapse RRMS primes healthy control (HC) neutrophils for more intensive ROI production after PMA stimulation in Bursttest analysis. (B) Contrary to RRMS, preactivation of HC neutrophils by NMOSD CSF is depended on C5a. Histograms depict mean intensity of ROI production ± SD taken from four independent experiments. (C) Ex vivo analysis of ROI production in circulating neutrophils in the patients with NMOSD, RRMS, OND, and HC reveals no differences in non-stimulated neutrophils, while following stimulation with PMA neutrophils from active NMOSD and relapse RRMS produce higher amounts of ROI. Data are presented as the means ± SD. [file image_4.tif]
