# Supplementary material for: C5a-Preactivated Neutrophils Are Critical for Autoimmune-Induced Astrocyte Dysregulation in Neuromyelitis Optica Spectrum Disorder
Source: Front Immunol. 2018 Jul 23;9:1694. doi: 10.3389/fimmu.2018.01694 (PMC6065055; doi:10.3389/fimmu.2018.01694)
Supplement: Table S1 — C5a-preactivated healthy control (HC) neutrophils polarize astrocytes into proinflammatory cells. Statistically significant differences between (a) astrocytes vs. astrocytes + neuromyelitis optica spectrum disorder (NMOSD) serum and (b) astrocytes + NMOSD serum vs. astrocytes + NMOSD serum + HC neutrophils. Data are presented as the means ± SEM from four independent experiments. [file table_1.PDF]

Supplementary TABLE 1. C5a preactivated HC neutrophils polarize astrocytes into proinflammatory cells. Statistically significant differences between: (*a*) astrocytes *vs*. astrocytes + NMOSD serum; (*b*) astrocytes + NMOSD serum *vs*. astrocytes + NMOSD serum+ HC neutrophils. Data are presented as the means ± S.E.M. from four independent experiments.

[pg/mL]	astrocytes	astrocytes + NMOSD serum	astrocytes + NMOSD serum + HC neutrophils	*p*<0.05
**IL-1β**	<0.04	1.6±1.19	9.7±5.95	*a, b*
**IL-1ra**	<0.15	1.1±0.76	13.7±7.09	*a, b*
**IL-2**	<0.3	4.9±2.87	23.8±7.98	*a, b*
**IL-4**	< 0.5	< 0.5	< 0.5	
**IL-5**	<0.15	<0.15	<0.15	
**IL-6**	50.4 ± 23.56	85.8±56.69	44.7±34.98	
**IL-7**	<0.2	<0.2	<0.2	
**IL-8 (CXCL8)**	131.9 ± 87.90	558.5±127.73	937.3±205.45	*a, b*
**IL-9**	12.1±8.98	16.8±9.40	13.3±8.38	
**IL-10**	<0.6	1.42±1.04	<0.6	
**IL-12(p70)**	<1.4	5.7±4.86	3.2±2.87	
**IL-13**	<0.1	<0.1	<0.1	
**IL-15**	3.9 ± 2.99	6.4±5.32	10.36±7.30	
**IL-17**	2.1 ± 0.87	4.7±3.49	76.3±12.40	*b*
**Eotaxin (CCL11)**	2.9 ± 1.76	5.3±4.37	9.8±5.69	
**FGF basic**	44.9 ± 12.32	46.3±18.63	241.8±93.29	*b*
**G-CSF**	<0.8	2.38±1.87	21.23±9.72	*b*
**GM-CSF**	54.6 ± 21.94	59.9±22.29	161.8±34.94	*b*
**IFN-γ**	<0.1	6.9±3.95	53.4±12.60	*a, b*
**IP-10 (CXCL10)**	5.8 ± 4.78	9.1±8.50	13.9±11.93	
**MCP-1(CCL2)**	133.4 ± 33.32	314.5±58.29	73.7±12.55	*a, b*
**MIP-1α (CCL3)**	<0.1	9.4±1.48	35.4±5.23	*a, b*
**PDGF-BB**	<1.4	59.2±18.89	109.1±32.34	*a, b*
**MIP-1β (CCL4)**	<0.1	5.5±1.12	11.9±3.21	*a, b*
**RANTES (CCL5)**	4.5 ± 2.33	3812.9±899.29	330.6±113.28	*a, b*
**TNF-α**	8.0 ± 7.51	24.4±9.23	122.4±55.25	*a, b*
**VEGF**	12.8 ±7.11	31.96±19.84	<2.4	*a, b*
